# Fluidization and Application of Carbon Nano Agglomerations

**DOI:** 10.1002/advs.202306355

**Published:** 2023-12-19

**Authors:** Sibo Chen, Yaxin Jiang, Zhenxing Zhu, Qi Zhang, Chenxi Zhang, Qiang Zhang, Weizhong Qian, Shijun Zhang, Fei Wei

**Affiliations:** ^1^ Beijing Key Laboratory of Green Chemical Reaction Engineering and Technology Department of Chemical Engineering Tsinghua University Beijing 100084 China; ^2^ Beijing Research Institute of Chemical Industry SINOPEC Beijing 100013 China; ^3^ Ordos Laboratory Inner Mongolia 017000 China

**Keywords:** agglomerate, carbon nanotubes, fluidization, nanocomposites, particle

## Abstract

Carbon nanomaterials are unique with excellent functionality and diverse structures. However, agglomerated structures are commonly formed because of small‐size effects and surface effects. Their hierarchical assembly into micro particles enables carbon nanomaterials to break the boundaries of classical Geldart particle classification before stable fluidization under gas‐solid interactions. Currently, there are few systematic reports regarding the structural evolution and fluidization mechanism of carbon nano agglomerations. Based on existing research on carbon nanomaterials, this article reviews the fluidized structure control and fluidization principles of prototypical carbon nanotubes (CNTs) as well as their nanocomposites. The controlled agglomerate fluidization technology leads to the successful mass production of agglomerated and aligned CNTs. In addition, the self‐similar agglomeration of individual ultralong CNTs and nanocomposites with silicon as model systems further exemplify the important role of surface structure and particle‐fluid interactions. These emerging nano agglomerations have endowed classical fluidization technology with more innovations in advanced applications like energy storage, biomedical, and electronics. This review aims to provide insights into the connections between fluidization and carbon nanomaterials by highlighting their hierarchical structural evolution and the principle of agglomerated fluidization, expecting to showcase the vitality and connotation of fluidization science and technology in the new era.

## Graphical abstract

This review highlights the fluidized structure control and fluidization principles for carbon nanotubes in various fractal agglomerations. The aim is to attract attention to the surface structure and fluidic properties in nano‐agglomerated fluidization and provide more insights into the connections between fluidization and carbon nanomaterials by highlighting their hierarchical structural evolution and the principle of nano‐agglomerated fluidization.

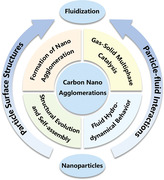

## 1 Introduction

**Figure 1:**
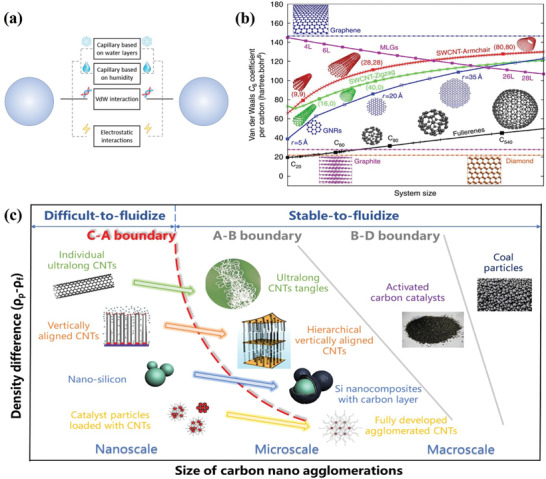
a) Forces acting between particles, including capillary based on water layers and humidity, vdW interaction, and electrostatic interaction. Reproduced with permission.^[^ ^19^ ^]^ Copyright 2023, Wiley. b) Scaling laws for vdW coefficients in various carbon nanomaterials. Reproduced with permission.^[^ ^17^ ^]^ Copyright 2013, Springer. c) The phase diagram of particle types based on carbon nano agglomerations is adapted from the classical Geldart particle classification in the fluidization (as shown in Figure 4d), which divides particles into four groups (A, B, C, and D) by the particle size and density difference with fluid. Activated carbon catalysts (Group‐B) and coal particles (Group‐D) are common stable‐to‐fluidize media, while nanosized Group‐C particles are difficult to fluidize. In this adapted diagram, the Group‐C particles including individual ultralong CNTs, vertically aligned CNTs, nano‐silicon and catalyst particles loaded with CNTs are respectively transformed into ultralong CNTs tangles, hierarchical vertically aligned CNTs, Si nanocomposites with carbon layer and fully developed agglomerated CNTs. The hierarchical assembly into micro particles enables carbon nanomaterials to break the C‐A boundary before stable fluidization under gas‐solid interactions. Reproduced with permission.^[^ ^20^, ^21^, ^22^, ^23^, ^24^ ^]^ Copyright 2021, 2011, 2018, 2003, 2010, The American Chemical Society, Wiley, Elsevier, Elsevier, Elsevier.

Nanomaterials have unique physicochemical properties due to their small size and large specific surface area. Their span can include multiple dimensions, such as 0D nanoparticles (carbon nanodots,^[^ ^1^, ^2^, ^3^ ^]^ noble metal nanoparticles,^[^ ^4^, ^5^ ^]^ inorganic quantum dots^[^ ^6^, ^7^ ^]^), 1D nanomaterials (nanotubes,^[^ ^8^, ^9^ ^]^ nanowires^[^ ^10^, ^11^ ^]^) and 2D nanofilms (graphene,^[^ ^12^, ^13^ ^]^ graphdiyne^[^ ^14^ ^]^). Because nanomaterials are located in the transition region between micro‐molecules and macroscopic condensed matter, they belong to a mesoscopic system with significant surface effect, small size effect, and quantum tunneling effect.^[^ ^15^, ^16^ ^]^ At the same time, when the material is reduced to the nanoscale, the interactions between particles increase sharply, mainly including the van der Waals (vdW) force, electrostatic interactions, etc. **Figure** **1**a shows a typical classification of interactions between particles, and vdW interaction plays an important role in nanomaterials' self‐assembly and functionalization processes. It is found that small fullerenes and graphene have the lowest and highest vdW coefficients respectively (Figure 1b), which is mainly due to structural polarization. Meanwhile, the vdW coefficient of single‐walled carbon nanotubes (SWCNTs) grows superlinearly as a function of the SWCNT radius. The coefficients are also chirality dependent, generally increasing faster for armchair nanotubes than for zigzag ones. This special scaling law leads to abnormal binding behavior between nanostructures of different dimensions, which helps control the self‐assembly of complex nanostructures.^[^ ^17^ ^]^ And in the same dimension, the high surface activity of nanomaterials and the strong interaction between particles make them easier to agglomerate,^[^ ^18^ ^]^ thus forming larger‐scale nano agglomerations of different structures and bringing differentiated macroscopic properties. However, the formation of nano agglomerations is commonly considered detrimental to the dispersion of materials and has not caught enough attention for a long time. Until recent years, agglomerations formed during fluidization were found to be a bridge from molecular assembly toward mass production of CNTs, which brings fresh perspectives and values to the study of nano agglomerates.

As a good gas‐solid mixing system, particle fluidization is a continuous process with high efficiency of heat and mass transfer on the macroscale. Based on this development, fluidization is a promising engineering technology for scaling‐up production of materials in the field of powder preparation, processing, and heterogeneous catalytic reaction. Fluidization technology can transform static particles into a dense flow state similar to a fluid, which can realize more efficient heat and mass transfer on the material surface and contribute to uniform and stable gas‐solid reactions.^[^ ^25^ ^]^ The reaction equipment that fluidizes static particles by the interactions between fluid and particles is defined as a fluidized bed. The formation of stable fluidization in a fluidized bed depends on the size of solid particles in the gas‐solid system, which is usually micrometer scale. However, in terms of nanoparticles, the stronger interactions will promote their adhesion, which often makes the bed of nanoparticles in the fluidized bed agglomerated in various structures and difficult to be monodispersed. Different from the dense agglomerations, the fractal agglomerations are composed of hierarchical nano clusters with different sizes and self‐similar morphology. In this case, the size of nano agglomerations with loose pores can reach hundreds of microns, in the range of the smooth fluidization operation domain. Previous studies have also witnessed the agglomerations of CNTs,^[^ ^26^ ^]^ nano‐SiO_2_,^[^ ^27^ ^]^ and other difficult‐to‐fluidize nanomaterials under specific conditions. These agglomerated structures exhibit special hydrodynamic properties during fluidization, which play a miraculously positive role in the processing and treatment of powders especially in the nano size.

Although the phenomenon of nanoparticle agglomeration and fluidization has been discovered for a long time, it has not yet received enough attention until the popularity of mass production in the research of nanomaterials, which also signifies the necessary path from basic research to industrial applications. For the agglomerated fluidization process of nano‐powders, the key is not to weaken or eliminate agglomeration but to change the apparent flow properties of nanomaterials by reasonably controlling fractal agglomerations with similar configurations. As shown in Figure 1c, some macroscopic particles, such as activated carbon catalysts and coal particles, are considered to be stable‐to‐fluidize media. In Geldart's particle classification chart based on particle size and density difference, Group‐C particles are difficult to be stably fluidized. However, several nanomaterials' hierarchical assembly into micro particles enables them to break the C‐A boundary before stable fluidization. These typical carbon nano agglomerations represent an intimate connection between material self‐assembly and fluidization science. This review focuses on the structural evolution and structural control of nano agglomerations for some Group‐C particles, so as to transform them into stable‐to‐fluidize Group‐A particles. We summarize the principles and phenomena in the process of nano agglomeration and emphasize its importance in solving the problems of self‐assembly of CNTs with high aspect ratios and mass production of aligned CNTs. Meanwhile, recent achievements in the industrial preparation of carbon nanocomposites with fluidization technology were also discussed. The research on the structure‐activity relationship of nano agglomerations reveals the influence of key factors, such as surface structure and flow state, on the agglomerate fluidization, which is intended to enlighten researchers on the fluidization preparation of nanomaterials with controlled structures.

## 2 Principle of Agglomeration and Fluidization

### 2.1 Formation of Nano Agglomeration

**Figure 2:**
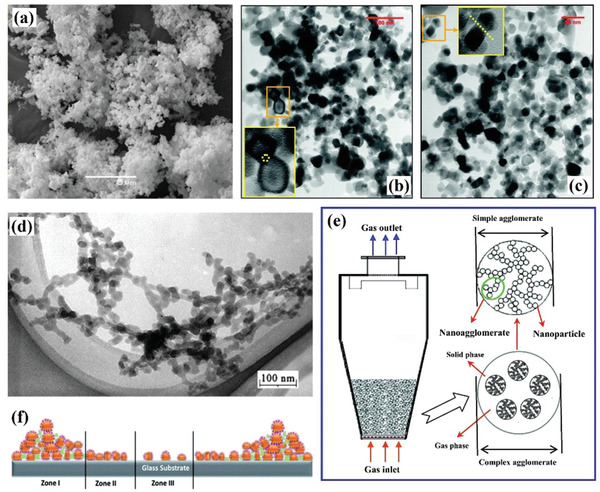
The formation of nano agglomerates and multi‐level structures. a) Typical SEM image of the nanocatalysts agglomerates. Reproduced with permission.^[^ ^32^ ^]^ Copyright 2022, Elsevier. b) TEM image of nanoparticles after fluidization and inset image indicates hard agglomerate structure. c) TEM image of nanoparticles after fluidization and inset image indicates soft agglomerate structure. b, c) Reproduced with permission.^[^ ^34^ ^]^ Copyright 2017, Elsevier. d) TEM image of chain‐like structure in nanoparticle agglomerates. Reproduced with permission.^[^ ^31^ ^]^ Copyright 2002, Elsevier. e) Schematic image of multi‐stage agglomerate of primary nanoparticles in a bench conical fluidized bed, including simple and complex agglomerates. Reproduced with permission.^[^ ^34^ ^]^ Copyright 2017, Elsevier. f) Distribution of virions on the substrates: Zone I represents a large agglomeration region of virions caused by the coffee ring effect, zone II depicts the close packing of virions along the surface, and zone III illustrates individual and agglomerated groups of virions bound to the surface during the incubation period. Reproduced with permission.^[^ ^44^ ^]^ Copyright 2020, The Royal Society of Chemistry.

The formation of agglomeration is a typical feature in the preparation of nanomaterials. In order to clearly define this concept, it is necessary to first understand the differences between agglomeration and aggregation. Agglomeration refers to the formation of relatively fragile clusters of nanoparticles in various structures that are attracted together through weak vdW force. Aggregation refers to the phase change and atomic surface diffusion of nanoparticles under high temperature or pressure, and a stable combination is achieved through the solid bridge or formation of a homogeneous phase, such as the Ostwald ripening of metal nanoparticles.^[^ ^28^ ^]^ The distinct difference between agglomeration and aggregation is whether it is easy to be re‐split into primary particles. In addition, due to the inter‐particle forces, aggregates can also form clusters during fluidization, leading to the formation of agglomerations.^[^ ^19^ ^]^ On the other hand, nano agglomerations can be divided into hard agglomerations and soft agglomerations based on the internal interconnected gas network caused by the tightness and fractal of the agglomeration. Typically, nanoparticles prepared by the vapor deposition method are loosely arranged soft agglomerations.^[^ ^29^, ^30^, ^31^, ^32^ ^]^ In contrast, nanoparticles prepared by chemical solution methods without sufficient surfactants or strong solid bridge connections are compact hard aggregates.^[^ ^33^, ^34^ ^]^ **Figure** **2**a shows nanoparticle agglomerates with a loose structure, while Figure 2b,c shows hard and soft agglomerates in nanoparticles respectively. Hard agglomerates are characteristic of non‐friable, coarse particles that are difficult to disperse and identified by the connection of spherical or near‐spherical particles (Figure 2b, inset), whereas soft agglomerates are friable, easily dispersed and identified via the approach of spherical or near‐spherical particles to each other (Figure 2c, inset).^[^ ^34^ ^]^ Different agglomerating modes directly determine the surface structure, fractal, density, and stability of particles, and affect the macroscopic state of nanomaterials. It is worth noting that soft agglomerations have a significantly reduced density due to their loose and porous internal structure, which is more conducive to fluidization in gas–solid two‐phase systems.

Surface physics and chemistry reveal that the formation of nano agglomerations results from the positive surface free energy of nanoparticles, which drives the spontaneous broadening of nanoparticle size distribution.^[^ ^35^ ^]^ For solid particles, increasing surface area requires overcoming intermolecular attraction and producing work on the system, which can be achieved by crushing and refining. In this process, the introduction of surface work increases the positive surface free energy of refined particles. The second law of thermodynamics points out that the spontaneous process at constant temperature and pressure always goes in the direction of reducing free energy. Because of the non‐fluidity of the surface atoms of solid particles, it is impossible to reduce the surface energy by changing the shape like liquid. The only way is to spontaneously form large agglomerated particles by interacting with adjacent particles around the environment.

### 2.2 Structural Evolution of Nano Agglomeration

The diversity of solid nanoparticles and the presence of various binding interactions result in different structures of nano agglomerations, which exhibit a trend of gradual evolution during the agglomeration process.^[^ ^36^, ^37^, ^38^ ^]^ In many years of fluidization research, researchers have found that the loose structure formed by particle agglomeration has a multi‐level evolution process. As shown in Figure 2d, spherical nano‐sized primary particles can be presented in the chain‐like connections,^[^ ^31^ ^]^ which can be influenced by the interactions of adhesion and solid bridge force. Particularly, adhesion is caused by the electrostatic force, vdW force, and other forces between the original nanoparticles,^[^ ^39^, ^40^ ^]^ while the solid bridge force is caused by the melting and coalescence.^[^ ^41^, ^42^ ^]^ Solid bridge force can ensure the stability of the loose 3D chain branch structure and avoid the breakage of the original nanoparticles under the high‐velocity gas flow. However, the dendrite structure is not the final form of particle fluidization but the more complex cluster structure formed by entanglement and interpenetration between the dendrites of particles. The mechanical strength of simple clusters is relatively low because they are only the primary interpenetration of dendritic structures. It will further self‐assemble and agglomerate to form a larger complex cluster until the formation of final fluidizable units. Figure 2e reveals the forms of complex and simple agglomerates in a fluidized bed, and reflects the formation of internal chain‐like structures inside the particles. During the fluidization, the surface structure of complex clusters will peel off and re‐adhesion, but the overall structure is in a state of dynamic equilibrium.

Considering that the solid bridge force between particles is not universal, which is usually affected by the melting point of the nanoscale‐inorganic matter, the characteristics of the particles, such as the surface structure, should be considered further in the agglomeration between nanoparticles. Low‐dimensional nanoparticles can have rich surface structures, such as coronavirus in nature. Taking the SARS‐CoV‐2 virus as an example, it has an average diameter of 100 nm, and its surface is adorned with spinous proteins measuring ≈20 nm in length.^[^ ^43^ ^]^ This radial surface structure has endowed the virus with low density and light mass, enabling the virus to float freely. Simultaneously, this morphology also provides more tentacle‐like structures with a larger specific surface area that promotes the virus to agglomerate for efficient spreading (Figure 2f).^[^ ^44^ ^]^ Similar to the agglomeration of 0D viruses, 1D materials represented by CNTs can also work as the tentacles of radiative agglomerates centered around the catalyst particles. At first, there was a strong interaction between CNTs and powder catalysts, which could form sub‐block structures with a diameter approaching 1 µm. After that, due to the vdW force and the entanglement between CNTs, sub‐clusters will further gather, even reaching 400–500 µm agglomerations.^[^ ^45^ ^]^ Xiang et al.^[^ ^46^ ^]^ also demonstrated that the bulk synthesis of CNT arrays can be achieved through the ferrocene chemical vapor deposition (CVD) process on spherical particles, resulting in a significantly reduced macroscopic particle density. This leads to weakened viscous interaction between particles and favorable fluidization conditions. In the subsequent chapters, the particle interaction and controllable fluidization of CNTs during the structural evolution process are further emphasized. To summarize, the ultimate result of hierarchical structural evolution during nanomaterial agglomeration is a decrease in overall particle surface energy and entry into a fluidizable state. As nanoscience continues to advance, further research on surface structure – an aspect traditionally overlooked by fluidization theory – will prove increasingly valuable.

### 2.3 Fluidization Mechanism of Nano Agglomerations

**Figure 3:**
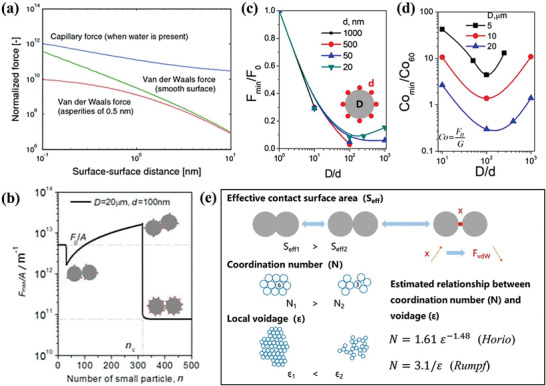
a) The main force between two nanoparticles is a function of the interparticle distance. The vdW force is related to the surface‐surface distance and roughness. Reproduced with permission.^[^ ^47^ ^]^ Copyright 2012, Springer. b) The relationship between the cohesive force and the number of small particles added, indicating that the interaction among particles can be mediated by surface coating. Reproduced with permission.^[^ ^25^ ^]^ Copyright 2016, Elsevier. c) The relationship between the interparticle force and the size ratio between the center particle and the ultrafine particle. d) The dependence of the cohesive number *C*_o_ on the size ratio between the center particle and the ultrafine particle, indicating that the central particles coated with ultrafine particles can regulate the interparticle force. e) Schematic of effective contact surface area, coordination number, and local voidage, which represents the effect of structural control on interparticle forces. The increase of *x* changes the effective contact surface area and will decrease interparticle *F*_vdW_. Meanwhile, the particles will be able to better fluidize in large local voidage with small coordination numbers.

For classical fluidization science, particle structure and properties are the key factors during the formation of stable multiphase flow. Especially in the gas‐solid reaction, the vdW force plays an important role in the agglomeration and flow behavior of particles, ignoring the capillary force affected by water. As shown in **Figure** **3**a, vdW force is related to the surface‐surface distance and roughness,^[^ ^47^, ^48^ ^]^ which can be expressed as:

(1) $$F_{\mathrm{vdW}}=\frac{A_{H}d_{p}^{3}}{12{\left(x+r_{\mathrm{asp}}\right)}^{2}{\left(x+r_{\mathrm{asp}}+d_{p}\right)}^{2}}$$

where *A*_H_ is the Hamaker constant, *d*_p_ is the diameter of particles, *x* is the surface‐surface distance and *r*_asp_ is the asperities of size. The formula can also be simplified as:

(2) $$F_{\mathrm{vdW}}=\frac{A_{H}d_{p}}{12x^{2}}$$

Because the vdW force is an interparticle cohesive force, it becomes the main reason that makes the fine particles difficult to be fluidized.^[^ ^49^ ^]^ Zhang et al.^[^ ^25^ ^]^ further analyzed the cohesive relationship between two rough spheres and found that although the surface covered with nanoparticles will initially lead to an increase of the cohesion force between particles, with the improvement of the coverage degree, it will result in the growth of the surface‐surface distance and then the interparticle cohesion force will decrease significantly (Figure 3b). Rough spheres can be regarded as ultrafine nanoparticles with uniform sizes covered by smooth spheres. The surface roughness of the particles will increase with the number of nanoparticles. It can be seen from Equation 1 that the increase of *r*_asp_ will be accompanied by the decrease of *F*_vdW_, and finally improve the fluidification performance. Zhu et al.^[^ ^50^, ^51^ ^]^ also proposed the “nanoparticle modulation” technique, which reduces the cohesion of fine particles and improves their flow and fluidization quality by applying ultrafine nanoparticles as fluidization additives. On the other hand, Huang et al.^[^ ^52^ ^]^ analyzed the influence of the surface roughness on interparticle forces, and the control over interparticle forces can improve the fluidization performance of particles. In the dry gas‐solid reaction system, the fluidization characteristics of particles depend on the cohesive force (mainly the vdW force) and gravity, and the ratio of the cohesive force to gravity can reflect the relative strength of the cohesive force of particles, which can be defined by the dimensionless cohesive number *C*_o_. It can be seen from Figures 3c,d that for central particles (diameter *D*) coated with ultrafine nanoparticles (diameter *d*), there is an extreme value of interparticle force with the change of *D*/*d*, which means that there is an optimal nanoparticle size for a definite diameter of the central particle. This is due to the fact that when *D*/*d* increases beyond a certain threshold, the surface of the central particles will be approximately flat for ultrafine nanoparticles. And the number of ultrafine nanoparticles that are actually exposed will rise sharply, resulting in the increase of the cohesive number *C*_o_^[^ ^52^ ^]^ Gu et al.^[^ ^53^ ^]^ highlight the effect of effective contact surface area *S*_eff_ and coordination number *N* on particle fluidization. The particles will be packed tightly on the condition of larger *N* and smaller local voidage *ε* (Figure 3e). Coordination number represents the number of particles directly adjacent to other particles, and Horio and Rumpf have also estimated the magnitude of coordination number, which is inversely proportional to the local voidage. On the other hand, the introduction of ultrafine particles can be seen as the spacing of *x* on the surface of particles, which will reduce the effective contact surface area. Equation 2 shows that the increase of *x* will weaken the interparticle *F*_vdW_. This factually emphasizes the importance of structure control in nano agglomerations. By controlling the particles' coordination structures, reducing the coordination number, and increasing the local voidage, it can also reduce the interparticle force and strengthen the fluidization.

**Figure 4:**
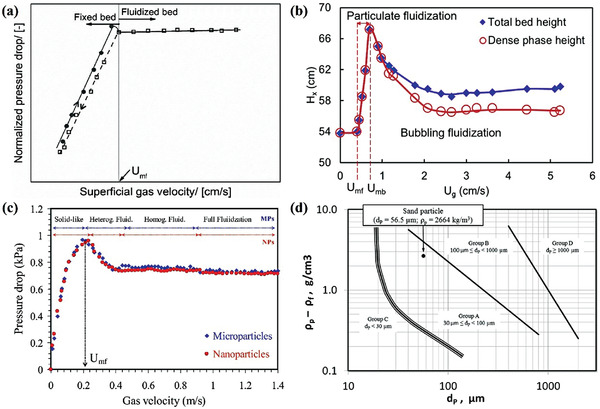
a) Typical curve of the normalized pressure drop in fluidized beds, where *U*_mf_ reflects the transition between fixed bed and fluidized bed. Reproduced with permission.^[^ ^54^ ^]^ Copyright 2022, Elsevier. b) Typical expansion curves for fluidizable powders. Reproduced with permission.^[^ ^62^ ^]^ Copyright 1986, Wiley. c) The bed pressure drop curves with a velocity of microparticles and nanoparticles. Reproduced with permission.^[^ ^34^ ^]^ Copyright 2017, Elsevier. d) Geldart particle classification phase diagram. Left axis: ρ_p_–ρ_f_ indicates the difference of density between the fluidizing media and gas; right axis: d_p_ indicates the particle diameter of the fluidizing media. Reproduced with permission.^[^ ^63^ ^]^ Copyright 2017, Elsevier.

In addition, fluidization is a motion phenomenon generated when solid particles and fluids come into contact. It involves complex gas‐solid interactions, and the exploration of the fluidization mechanism cannot ignore the motion behavior of fluids. The most important parameter of macroscopic fluid movement is the gas velocity in a gas‐solid fluidization system. For reactors loaded with solid particles, as the fluid passes from bottom to top, the pressure drop of the fluid will change as the flow rate increases.^[^ ^54^ ^]^ **Figure** **4**a shows that the pressure drop increases as the flow rate rises, indicating the fixed bed state at a low flow rate. When the flow rate increases to a certain value, it comes to a balance between the drag force of the fluid on the particles and the particle gravity, so that the particles enter a fluidized state from a stationary state. The particle bed porosity increases and the pressure drop almost remains constant.^[^ ^54^ ^]^ This gas velocity is the critical fluidization velocity (*U*_mf_, the so‐called minimum fluidization velocity^[^ ^55^ ^]^). The *U*_mf_ reflects the strength of particle fluidization ability, which is usually determined by the properties of the particles themselves and the structure of the fluidized bed. In early studies, Geldart,^[^ ^56^ ^]^ Crandfield,^[^ ^57^ ^]^ Saxena and Jadav^[^ ^58^ ^]^ have quantitatively demonstrated that *U*_mf_ is a function of bed mass or height. Caiedo et al.^[^ ^59^ ^]^ further conducted fluidized bed experiments with different particle sizes in a 2D fluidized bed to reveal the dependence of bed friction on the *U*_mf_, indicating that the *U*_mf_ depends on the bed height, particle size, and column width. Thus, the *U*_mf_ is an important parameter for studying the interaction between particles and fluids in fluidization science.

Figure 4b shows the bed expansion curve of typical fluidizable powders, with bubbles formed at the minimum bubbling velocity (*U*_mb_). The powders can change from particulate fluidization to bubbling fluidization when the gas velocity goes beyond the critical value *U*_mb_, and the total bed height and dense phase height show an opposite trend from before. During the particulate fluidization, the gas has intimate contact with the solids, which can be treated as a homogeneous system.^[^ ^60^ ^]^ The appearance of bubbles breaks this homogeneous state, thus the total bed height and dense phase height gradually decrease and eventually reach the dynamic equilibrium. Figure 4c compares the bed pressure drop curves between microparticles and nanoparticles.^[^ ^34^ ^]^ At the minimum fluidization velocity, the bed pressure drop reaches an extreme value and then decreases to a stable range with the increase of the gas velocity. Although the primary sizes of nanoparticles and microparticles differ by nearly three orders of magnitude, they still exhibit similar fluidization behavior, mainly due to the formation of nano agglomerations. This means that even though nanoparticles are regarded as traditional difficult‐to‐fluidize particles, homogeneous fluidization can be achieved with changes in particle structure.

The classification of particle fluidization can be traced back to the Geldart phase diagram.^[^ ^61^ ^]^ As shown in Figure 4d, Group‐A particles exhibit excellent initial fluidization ability due to their small original particle size, typically in the range of 30–100 µm, and their tendency not to spontaneously agglomerate. For instance, sand particles with a diameter of 56.5 µm are categorized as Group‐A particles, which typically exhibit a uniformly dispersed fluidization zone and high bed expansion rate before bubbling gas velocity. Compared with Group‐B particles (average size is 100–1000 µm) and Group‐D particles (size ≥ 1000 µm), the Group‐A particle bed collapses more slowly after stopping the gas supply. It is facile to establish coalescent fluidization with large bubbles, which possess the optimal fluidization quality. High‐quality fluidization manifests complete and uniform contact and reaction opportunities between the particles and the fluid, which guarantees elevated mass and heat transfer efficiency as well as the appropriate residence time of the fluid throughout the bed.

The exceptional properties of Group‐A particles have rendered them wide attention and research focus among scholars. In the past, extensive investigations have been conducted on particle and gas properties (such as particle‐wall friction coefficient, interparticle forces, and density),^[^ ^64^ ^]^ particle size distribution,^[^ ^54^ ^]^ and temperature.^[^ ^65^ ^]^ Unlike the other three kinds of particles, nanomaterials belong to Group‐C ultrafine particles, and these primary nanoparticles exhibit significant resistance to fluidization. Despite that, Group‐C particles are increasingly used in the modern manufacturing industry, such as carbon aerogel (diameter ≈200 nm) in industrial capacitors, as well as activated carbon electrode particles and graphite in negative electrode (diameter 5–6 µm). Due to their tight packing and strong interactions in the reactor, Group‐C particles are prone to consolidation in the particle bed. When the fluid flows through the bed, it often escapes through the large gaps between those tightly bound nanoparticles. It commonly causes gas flow short‐circuiting, which seriously hinders efficient heat and mass transfer between gas‐solid phases. When the gas velocity falls within an appropriate range, the initially consolidated particle bed is fragmented into small agglomerates by the shear force of the gas flow. The formation of these agglomerates signifies the emergence of fluidizable units, which constitutes a primary factor in achieving initial fluidization.

The formation of agglomerates provides crucial conditions for investigating the fluidization behavior of fine particles, thereby rendering it significant to explore the fluidization characteristics of nanomaterials. Previous studies^[^ ^26^ ^]^ revealed the occurrence of Geldart‐like agglomerate particulate fluidization in the mass production of CNTs. Although CNTs exhibit a smooth and highly expanded fluidization, a strong hysteresis still exists in their fluidization. On the defluidization branch, CNTs are similar to Group‐A particles, but the weak interaction and highly porous structure of the agglomerates make their fluidization more special. Lee et al.^[^ ^66^ ^]^ also studied the fluidization behavior of different multi‐walled carbon nanotubes (MWCNTs) and compared them with Geldart‐classified particles. Their research indicated that the fluidization behavior of MWCNT agglomerations formed by cohesive forces is similar to that of Group‐A particles. Low‐density agglomerations have a high minimum fluidization velocity and bed expansion ratio.

**Figure 5:**
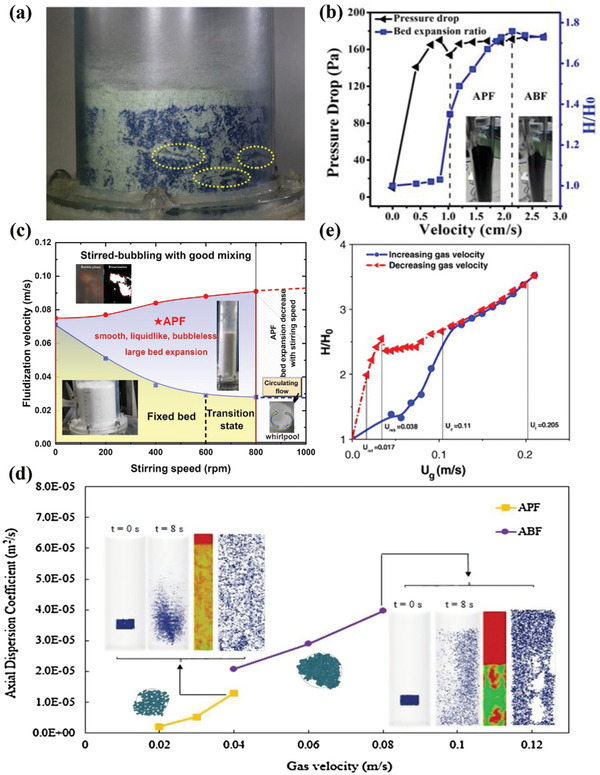
a) Channel‐like structures in a bed of binary particles. Reproduced with permission.^[^ ^69^ ^]^ Copyright 2014, Elsevier. b) The fluidization curve of the fluidized bed with agglomerates of 150–200 µm as bed materials. The inset is the pictures of the fluidized bed in APF and ABF. Reproduced with permission.^[^ ^77^ ^]^ Copyright 2014, Elsevier. c) Flow regimes of cohesive agglomerated powders with stirring assistance. Reproduced with permission.^[^ ^81^ ^]^ Copyright 2023, Elsevier. d) Simulation of solid mixing in nanoparticle aggregation fluidized bed using CFD‐DEM research. Reproduced with permission.^[^ ^87^ ^]^ Copyright 2021, Elsevier. e) Dependence of bed expansion on gas velocity in a CNT nano‐agglomerate fluidized bed. Reproduced with permission.^[^ ^26^ ^]^ Copyright 2006, Wiley.

In particle bed destruction, researchers have widely acknowledged that the formation of nano agglomerates is influenced by the vdW force, which is the most prevalent interparticle force, in conjunction with gas velocity.^[^ ^67^, ^68^ ^]^ As the main reason for the self‐agglomeration of nanoparticles, vdW interaction is not simply the adhesion of a pair of particle clusters. Rather, when many particles are present, spontaneous clustering occurs and leads to deformation and uneven contact areas among particles. This uneven packing structure results in agglomerates with varying local porosity *ε*, leading to low bulk density and the formation of porous agglomerations. The loose space structure and multi‐level agglomeration effectively weaken the cohesive force between ultrafine particles, preventing the bed from hardening. At the same time, the relatively low density also weakens the gravitational effect, facilitating agglomerations to float in the airflow. The fixed bed can experience unstable ditch flow when affected by initial airflow. As gas velocity gradually increases, clusters in local areas will be fluidized. The collision and cohesive force between particles result in the division of the bed, expansion of fluidization area, and ultimately uniform fluidization throughout the entire bed. As shown in **Figure** **5**a, the channel structure present in the initial particle bed layer can impede particle separation in binary particle systems.^[^ ^69^ ^]^ The occurrence of this phenomenon should be minimized during the actual fluidization process to facilitate the formation of nano agglomerates and ensure stable particle fluidization.

### 2.4 Characteristics of Nano Agglomerate Fluidization

#### 2.4.1 Agglomerate Particulate Fluidization

The formation of nanoclusters gives Group‐C particles a chance to surpass the scale limit and become fluidizable Group‐A or B particles, exhibiting different fluidization behaviors. Certain nanoparticles exhibit limited bed expansion during fluidization, resulting in the rapid passage of large bubbles through the bed, defined as agglomeration bubbling fluidization (ABF).^[^ ^31^, ^70^, ^71^ ^]^ On the contrary, the ultrafine particles follow agglomerate particulate fluidization (APF).^[^ ^31^, ^72^, ^73^ ^]^ During this process, the particle bed can attain remarkably high expansion and maintain stable fluidization without bubbles.^[^ ^73^ ^]^ Moreover, the airflow velocity as a function of voidage around the fluidized agglomerates follows the Richardson–Zaki equation.^[^ ^31^, ^55^, ^74^ ^]^ Navid et al.^[^ ^75^ ^]^ investigated the fluidization behavior of hydrophobic nanoparticles at low temperatures and discovered their remarkable bed expansion, resulting in stable and uniform fluidization without bubble formation. Conversely, hydrophilic nanoparticles exhibit APF behavior at high temperatures due to temperature‐induced changes in hydrogen bonding and vdW force between particles. The analysis of coherent and incoherent pressure fluctuations has also been utilized to determine the behavior of bubbles and agglomerates and to unveil temperature‐induced alterations in particle fluidization patterns. Asif et al.^[^ ^76^ ^]^ studied the kinetics of fluidized beds containing agglomerated hydrophobic nanoparticles with APF behavior and observed that the velocity of the fluidized gas undergoes regular step changes. On the contrary, in the fluidization process of particle agglomerates, a complete adherence to a single fluidization mode is not observed, and changes in fluidization mode may occur as gas velocity varies. Wang et al.^[^ ^77^ ^]^ found that smaller agglomerates transitioned from APF to ABF behavior with changes in gas velocity. Figure 5b illustrates the corresponding fluidization curve. When the gas velocity exceeds 2.25 cm s^−1^, the bed height decreases, accompanied by pronounced fluctuations at the upper interface, and agglomerates enter the ABF mode. Meanwhile, to effectively regulate the fluidization behavior of agglomerates, various external factors such as vibration,^[^ ^78^, ^79^, ^80^ ^]^ high stirring speed,^[^ ^81^ ^]^ magnetic impaction,^[^ ^82^ ^]^ microjets,^[^ ^83^ ^]^ and others are widely employed to facilitate fluidization and mixing. Figure 5c illustrates the evolution of cohesive agglomerated powder flow states under varying stirring speeds, with an expanding APF operating range as both stirring speed and superficial gas velocity increase.

In addition to investigating the agglomerate fluidization behavior of single particles, researchers have also shown great interest in studying hybrid particle systems. Zhou's research team^[^ ^84^ ^]^ revealed that binary mixture nanoparticles can be effectively fluidized under vibrating conditions. The Richardson–Zaki index of single and binary mixed nanoparticles was calculated through linear regression, indicating that the APF performance can be significantly enhanced by incorporating hybrid nanoparticles. Another study^[^ ^85^ ^]^ suggests that the behavior of hybrid nanoparticles can be improved by adding coarse particles, which penetrate the agglomerates and alter their structure, thereby impeding their growth through collision or friction. This fluidization optimization measure based on particle design does not require equipment updates and has lower transformation costs. The principle of enhancing the fluidization behavior of nanoparticles by incorporating coarse fluid catalytic cracking particles lies in the formation of core‐shell structure,^[^ ^86^ ^]^ which leads to lower voidage, smaller roundness, and cohesion, rendering them superior to pure nano agglomerates in fluidization behavior. Reza et al.^[^ ^87^ ^]^ systematically studied the mixing behavior of nanoparticle agglomerates in APF and ABF regimes using the Computational Fluid Dynamics Discrete Element Method (CFD‐DEM) method (Figure 5d) and employed parallel programming with both computer GPU and CPU resources to compare the gas volume fraction and dispersion coefficients of APF and ABF particles at different gas velocities. The APF particles used for simulation are more porous and have a netlike structure, while the ABF particles have a compact structure. The simulation results show that the dispersion of APF particles is more uniform and the bed expansion is higher than ABF particles. The movement of bubbles in the ABF regime provides a larger driving force for the axial movement of agglomerates, so the axial dispersion coefficient is higher than that of APF particles. Additionally, they examined the Lacey mixing index in two fluidization behaviors to assess mixing quality and determined that ABF behavior yields superior results compared to APF behavior. These findings underscore the complexity of APF as a gas‐solid reaction system, necessitating further theoretical and experimental research for the industrial development of nanomaterials.

#### 2.4.2 Thixotropy and Hysteresis

Thixotropy and hysteresis are also significant characteristics in analyzing the APF behavior of native nanoparticles. Thixotropy reflects the particle flow transition during gas flow through a fixed bed, where particles near the ditch flow are initially fluidized before breaking up over a larger range. The corresponding gas velocity is transformed into the thixotropy gas velocity *U*_m_,^[^ ^88^ ^]^ which essentially equals the minimum fluidization velocity and reflects the transformation of the particle bed layer. In a conventional fluidized bed, uneven accumulation of particles usually leads to violent eruption through local channels accompanied by overpressure which is caused by pressure drop above the weight of particles per unit area. As the gas flow rate increases, the channels will merge and fluidization will first occur in the central area of the bed. Subsequently, this region expands toward the walls until complete fluidization is achieved throughout the entire bed.^[^ ^89^ ^]^ However, excessively high gas velocity will damage the original clear particle surface, making small agglomerates lift out and cause material loss. When the gas velocity is reduced to *U*_m_, the bed does not immediately stop fluidization but maintains a constant pressure drop within a certain range. This phenomenon is known as hysteresis and reflects the law of the bed layer falling back when the gas velocity decreases.^[^ ^55^, ^90^ ^]^ However, when the gas velocity decreases to the critical gas velocity *U*_a_ (the minimum gas velocity of maintaining agglomerate fluidization), the bed height will suddenly drop and transition into a channel flow state.^[^ ^88^ ^]^ Figure 5e shows the dependence of bed expansion of CNTs on gas velocity in a fluidized bed. When the gas velocity decreases, the bed expansion changes differently from the curve when the gas velocity increases. Before the minimum bubbling velocity, the bed expansion declined slowly, indicating a strong hysteresis of the CNT agglomerates.^[^ ^26^ ^]^ Thixotropy and hysteresis represent special phenomena in the fluidization process of nano agglomerates. Further research on these phenomena would be advantageous in comprehending the fluidization behavior of particles in gas‐solid reactions. Syed et al.^[^ ^63^, ^91^ ^]^ discovered that particle mixing‐assisted fluidization technology can effectively mitigate hysteresis effects when examining particle mixing. External particles with larger size and density collide with nano agglomerates, eliminating bed non‐homogeneities and effectively improving fluidization hydrodynamics.

## 3 Fluidization and Mass Production of CNTs

### 3.1 Agglomerated CNTs

**Figure 6:**
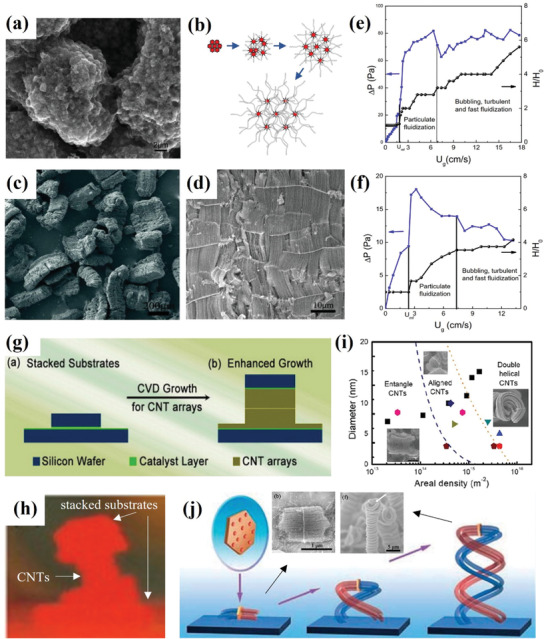
a) Agglomerate structure of MWCNTs. Reproduced with permission.^[^ ^45^ ^]^ Copyright 2008, Elsevier. b) Schematic mechanism of CNT agglomerate formation. Reproduced with permission.^[^ ^23^ ^]^ Copyright 2003, Elsevier. c) Macroscopic view of the vertically aligned CNT arrays grown by internal intercalation and present in a typical granular form. Reproduced with permission.^[^ ^96^ ^]^ Copyright 2009, Elsevier. d) SEM image of vermiculite‐CNTs composites after intercalation, showing an expanded layered structure comprising alternate aligned CNTs and vermiculite sheets. Reproduced with permission.^[^ ^97^ ^]^ Copyright 2009, Wiley‐VCH. e) The fluidization characteristic of the lamellar Fe/Mo/vermiculite catalyst. f) The typical fluidization characteristic of the CNT array particles. e, f) Reproduced with permission.^[^ ^96^ ^]^ Copyright 2009, Elsevier. g) Schematic illustration of silicon wafer stacked growth of aligned CNTs. h) The in situ recorded image for the growth of vertically aligned CNTs with the stacked substrates. g, h) Reproduced with permission.^[^ ^98^ ^]^ Copyright 2011, Elsevier. i) Phase diagram of the agglomerated CNTs. The hierarchical morphology alters with the areal density of CNTs, from CNTs that are entangled, to the ones that are aligned CNTs and to ones that are double helical. Reproduced with permission.^[^ ^100^ ^]^ Copyright 2021, Wiley‐VCH. j) Formation of the double‐helix CNTs. Inset, SEM image of the double‐helix CNTs at different growth stages. Reproduced with permission.^[^ ^101^, ^102^ ^]^ Copyright 2010, 2012 Wiley‐VCH, The American Chemical Society.

To better understand the significance of fluidization of nano agglomerations, CNTs are utilized as an example to demonstrate their important applications. The conventional method for CNT preparation involves growing them in a fixed bed within a horizontal tubular furnace. However, this process is hindered by uneven gas‐solid mixing, which severely limits CNT growth efficiency. Additionally, the dense accumulation of catalyst particles impedes the diffusion of reaction gases and dissipation of byproducts. In contrast, fluidization technology based on nano agglomeration can effectively address the issues above, as evidenced by its successful application in the mass production of agglomerated CNTs. **Figure** **6**a,b depicts a typical agglomerate structure of CNTs and a schematic of agglomerate formation, respectively. The original catalyst particles are first crushed by CNT growth, and then the catalyst sites are separated and form sub‐agglomerates, eventually forming fully developed agglomerates.^[^ ^45^ ^]^ As early as 2004, Windle et al.^[^ ^92^ ^]^ recognized the challenge of controlling the growth of SWCNTs due to the high‐temperature sintering of metal catalysts in a horizontal tubular furnace. However, by precisely regulating gas velocity and introducing SiO_2_ particles as nano‐catalyst carriers and fluidizable units, stable SWCNT growth can be achieved at lower temperatures. In 2008, Yang et al.^[^ ^93^ ^]^ transformed conventional MCM‐41 catalysts from Group‐C particles to Group‐A particles by regulating their diameters. Fluidization studies have demonstrated that larger MCM‐41 particles tend to be fluidized in agglomerates, exhibiting similar fluidization characteristics to those of particles and bubbles, which can be utilized for catalyzing SWCNT growth. In 2009, Harris et al.^[^ ^94^ ^]^ systematically studied the crucial factor of the ratio between superficial gas velocity and minimum fluidization velocity during the fluidization growth of MWCNTs. They discovered that as reaction time increased, pressure drop in the fluidized bed also increased. Meanwhile, the deposition of CNTs on catalyst particles caused significant changes to both the depth and structure of the fluidized bed, resulting in highly interwoven agglomerated CNTs.

### 3.2 Vertically Aligned CNTs

The vertically aligned CNTs are a specially ordered structure. The catalyst loaded on the carrier promotes the decomposition and assembly of carbon sources, forming a dense forest of vertically aligned CNTs. Unlike the fluidization of agglomerated CNTs, achieving a continuous and stable state during the fluidization process for vertically aligned CNTs requires greater care, as violent particle movement within the fluidized bed can lead to strong collisions, which will hinder the orderly vertical arrangement of CNTs. In the early stage, Wei et al.^[^ ^95^ ^]^ discovered that the length of CNT arrays grown on ceramic ball templates could reach up to 1100 µm through a cooperative growth mechanism, resulting in a radial flower‐like structure throughout the sphere. However, the particle diameter of the ceramic ball is indeed 700 µm and increased to 3 mm after CNT array growth, which is evidently beyond the scope of Group‐A particles. Fluidization in the form of a spouted bed will cause serious abrasion between particles.

Internal intercalation growth is a superior approach to fabricating vertically aligned CNTs compared with surface radiation growth, as it overcomes the challenge of maintaining CNT morphology during nano agglomerate fluidization and surface radiation growth. The utilization of layered inorganic materials provides support and a template for the vertically aligned CNTs, which will contribute to preserving the orientation and structure throughout the fluidization process. Wei et al.^[^ ^96^ ^]^ discovered that vermiculite can serve as an excellent template for the fluidization unit of vertically aligned CNTs. Vermiculite particles, with a diameter in the range of 100–300 µm, were obtained through crushing and screening. The Fe/Mo active component was loaded within the lamellar structures of the particles, resulting in catalyst particles with a bulk density of 160 kg m^−3^ and belonging to Group‐A particles. With ethylene as a carbon source, CNTs can grow steadily at active sites between layers (Figure 6c), exhibiting excellent alignment and high purity. The vermiculite is sandwiched by vertically aligned CNTs with uniform lengths, and the vermiculite sheets can be distinguished clearly along the perpendicular direction (Figure 6d).^[^ ^97^ ^]^ Figure 6e,f depicts the fluidization characteristics of the catalyst and vertically aligned CNTs. With the increase of gas velocity, the pressure drop increases first and then becomes stable, while the bed height keeps rising. The CNT products and vermiculite catalysts possess similar fluidization curves, both undergoing particle, bubble, and turbulence fluidization with increasing gas velocity.^[^ ^96^ ^]^ At a gas velocity of 7.0 cm s^−1^, stable fluidization of CNT products is achieved, providing significant advantages for mass production. Furthermore, this growth mode of internal intercalation induces an interlayer confined effect, enabling synchronous growth among individuals of vertically aligned CNTs while protecting them from collisions and structural damage during fluidization (Figure 6g). The real‐time observation in Figure 6h verified the enhanced growth of millimeter‐long vertically aligned CNTs between stacked substrates, with a maximum initial growth rate of 38 µm s^−1^, nearly fiftyfold faster than the normal growth.^[^ ^98^ ^]^ The high‐speed growth can be attributed to the template autocatalysis mechanism, where the elongated growth is a consequence of carbon atoms assembled under autocatalysis of the rim, while the complicated but temporary thermodynamic nucleation can be neglected. The rate‐determining kinetics reflect the interactions between CNT materials and the surrounding catalytic atmosphere.^[^ ^99^ ^]^ For example, the morphology of assembled CNTs can be determined by the areal density and diameter of catalyst nanoparticles (Figure 6i).^[^ ^100^ ^]^ When the areal density of nanoparticles is lower than 10^−14^ m^2^, the CNTs tend to agglomerate in a larger‐size particle, while the vertically aligned CNTs will dominate when the areal density increases to 10^−14^–10^−15^ m^2^. It will be more prominent when the areal density of nanoparticles goes beyond 10^−15^ m^−2^, where the vertically aligned CNTs on both sides of the template can be further assembled into a double‐helix form (Figure 6j).^[^ ^100^ ^]^ However, due to their exquisite structure, it can be more difficult to fluidize them in the form of Group‐A particles like the agglomerated or vertically aligned CNTs, so that their output and industrial application are still at a relatively immature level.

**Figure 7:**
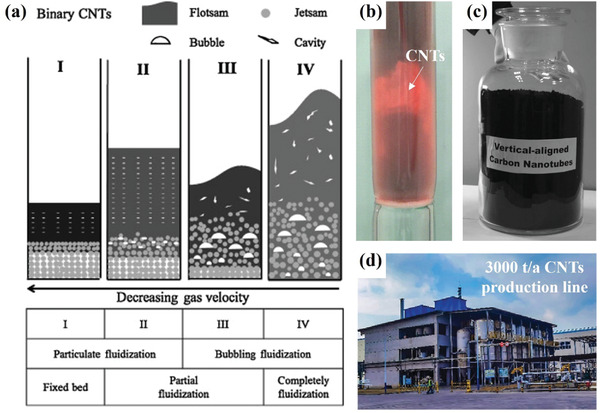
a) Conceptual diagram of fluidization characteristics of binary MWCNTs with decreasing gas velocity. Reproduced with permission.^[^ ^66^ ^]^ Copyright 2016, Elsevier. b) A photo of the actual fluidization of CNTs. c) A large amount of aligned CNTs grown on Fe/Mo/vermiculite catalyst. Reproduced with permission.^[^ ^96^ ^]^ Copyright 2009, Elsevier. d) Production line of aligned CNTs with an output of 3000 t/a. Reproduced with permission.^[^ ^100^ ^]^ Copyright 2022, Wiley‐VCH.

In fact, whether it is surface radiation growth or internal intercalation growth, the key to achieving fluidization of vertically aligned CNTs lies in attaining stable nano agglomerate fluidization. Agglomerated CNTs can form loose agglomerations and undergo the fluidization process after directly agglomerating by the cohesive force from their surface structure. The ordered CNT array structure has changed the original surface structure of the particles by introducing an additional supporting substrate, as a protective layer. At this time, appropriate gas velocity can ensure smooth fluidization of large particles and help maintain the alignment of internal CNTs. Based on similar concepts and novel technologies, recent advances have been made in the fluidization of vertically aligned CNTs. Wei et al.^[^ ^103^ ^]^ utilized Fe/Mg/Al layered double hydroxides ultrafine catalyst particles and introduced a uniform magnetic field into the fluidized bed reactor to achieve an orderly arrangement of catalyst sheets, resulting in the stable and sustainable growth of CNT bundles. Meanwhile, the fluidized bed is a reactor with multiple scientific research values. Lee et al.^[^ ^66^ ^]^ investigated the fluidization behavior of binary mixtures comprising various types of MWCNTs, elucidating the particle bed fallback phenomenon that occurs as gas velocity decreases. As shown in **Figure** **7**a, it was found that the bed height of the debris slightly increased from region III to II, as the irregular cavities observed in the bubbling fluidization of the debris were replaced with regular and smaller cavities. Their other research^[^ ^104^ ^]^ revealed that the bed collapse behavior of agglomerated multi‐walled CNTs is similar to Group‐C particles rather than Group‐A particles, as determined through hydrodynamic characteristics and size analysis. Figure 7b depicts an in situ observation of the fluidization preparation of CNTs, revealing a distinct flow bed layer formed by the floating airflow. The obtained vertically aligned CNTs are shown in Figure 7c. Furthermore, extensive research has been conducted on particle size,^[^ ^105^ ^]^ catalyst characteristics,^[^ ^32^ ^]^ and other factors involved in synthesizing CNT agglomerations in fluidized beds. He et al.^[^ ^106^, ^107^, ^108^ ^]^ also studied the fluidization characteristics and hydrodynamics of CNT particles in a conical fluidized bed reactor through an innovative fluidized bed reactor. More importantly, the industrialization of CNTs based on nano‐agglomerated fluidization has made breakthrough progress in recent years. Since the earliest mass production of agglomerated MWCNTs via fluidization in 2002,^[^ ^30^ ^]^ the highest MWCNTs yield has increased from 15 kg h^−1^ to over 116 kg h^−1^, and the highest SWCNTs yield has surpassed 8.6 kg h^−1^.^[^ ^100^ ^]^ A typical production line of aligned CNTs has been shown in Figure 7d with an annual output of 3000 t, which represents the outstanding progress of nano‐agglomerated fluidization in the mass production of CNTs, which is expected to bring quadrillions of times of increase in industrial output value. It is believed that combining fluidization theory with nanomaterial granulation strategies will guide future engineering mass production of materials, which can stimulate prosperous developments of advanced manufacturing and applications such as ultralight carbon aerogel,^[^ ^109^, ^110^ ^]^ high‐performance CNT fiber,^[^ ^111^ ^]^ advanced flywheel for energy storage,^[^ ^112^ ^]^ intelligent biomedical sensing,^[^ ^113^, ^114^ ^]^ electromagnetic shielding and emitting materials,^[^ ^115^, ^116^ ^]^ etc.

## 4 Structural Control and Fluidization of Horizontally Aligned CNTs

### 4.1 Intertube Coalescence of Horizontally Aligned CNTs

The preceding chapter has demonstrated the significant importance of nano agglomerate fluidization in macroscopic CNTs with varying structures, primarily addressing the issue of mass production. Traditional fluidization's agglomeration is not entirely advantageous for controlling material structure; its primary objective is to achieve fluidization through agglomeration while minimizing the influence on material properties. However, agglomeration offers the advantage of enhancing material agglomerate degree within localized confined spaces, which has aroused researchers' interest in horizontally aligned CNTs (HACNTs).^[^ ^20^, ^117^ ^]^ This is a dilute phase fluidization model in which the gas is the main flow body.

**Figure 8:**
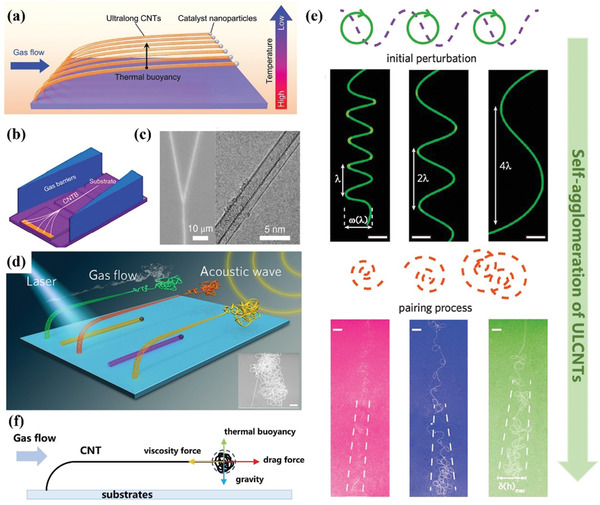
a) Schematic illustration of the kite‐flying growth mechanism of ULCNTs. Reproduced with permission.^[^ ^119^ ^]^ Copyright 2022, Wiley‐VCH. b) Schematic illustration of the in situ fabrication of CNT bundles by the GFF method. c) SEM and TEM images of CNT bundles consisting of two CNTs. b, c) Reproduced with permission.^[^ ^123^ ^]^ Copyright 2018, Springer. d) Schematic illustration of the synthesis of ULCNT agglomerates. Reproduced with permission.^[^ ^124^ ^]^ Copyright 2016, American Association for the Advancement of Science. e) Structural evolution of ULCNT tangles. ULCNTs undergo a transition from sinusoidal initial structure to agglomeration due to airflow vortex perturbation. Reproduced with permission.^[^ ^20^ ^]^ Copyright 2021, The American Chemical Society. f) Schematic illustration for force analysis of ULCNT tangles.

To understand the structural control issues in HACNTs, it is imperative first to analyze their growth process. HACNTs, especially ultralong CNTs (ULCNTs), are defect‐free materials with excellent mechanical, electrical, and thermal properties, which are obtained by guiding the horizontal airflow on the fixed substrate. Unlike agglomerated CNTs and vertically aligned CNTs, HACNTs follow the “kite mechanism” of tip growth within a horizontal stationary fluidized bed.^[^ ^118^ ^]^ Under the combined influence of airflow drag, viscosity, gravity, and thermal buoyancy, HACNTs exist in a floating state (**Figure** **8**a).^[^ ^119^ ^]^ With vapor‐assisted growth conditions, HACNTs can quickly reach decimeter‐level lengths and achieve an ultrafast growth rate of 80–90 µm s^−1^.^[^ ^120^, ^121^ ^]^ Because the gas‐solid two‐phase reaction experienced by HACNTs is based on horizontal flow, the force differs from that of the vertically aligned CNTs in the vertically fluidized bed. For HACNTs, the drag force and buoyancy of the airflow are balanced by gravity along the vertical direction, while the cohesive force between tube components dominates as the surface force. In horizontal airflow, the drag force is a surface force influenced by material surface area and gas velocity. Therefore, the gas flow has a significant impact on the structure and arrangement of HACNTs. The growth of HACNT arrays obeys the Schulz–Flory distribution, which was originally used to describe the relative ratios of polymers with different lengths after a polymerization process, and it has been proved effective in describing the exponential decay of ULCNTs density with length.^[^ ^118^ ^]^ CNTs with different bandgap structures will also exhibit a specific population decay due to the rate selection growth mechanism.^[^ ^122^ ^]^ In the initial high‐density region, the dense arrangement of CNTs and strong inter‐tube interactions make agglomeration behavior between tubes more susceptible to airflow effects, resulting in tube bundle formation. Bai et al.^[^ ^123^ ^]^ devised an in situ gas flow focusing (GFF) strategy for preparing ULCNT bundles (Figure 8b). This approach enables the coalescence and bunching of HACNTs through the vdW interaction between adjacent CNTs while preserving the stability of the original gas velocity. Figure 8c depicts the typical microstructure of the coalescence between two adjacent CNTs. When the CNT density is high, the drag force exerted on CNTs perpendicular to the direction of airflow is smaller than that of the vdW force, leading to the coalescence of ULCNTs into bundles. In particular, compared with the agglomerated and vertically aligned CNTs, the ULCNTs have the highest aspect ratio and specific surface energy and can form a tight‐contact bundle with ultra‐high strength associated with the strong vdW interaction.

### 4.2 Self‐Agglomeration of ULCNTs

In fact, there are two kinds of agglomeration for HACNTs fluidized systems. When the length is short, the vdW interaction is dominant, and the dense horizontally aligned CNTs tend to coalesce into bundles. Conversely, as the length increases, the high aspect ratio of ULCNTs also confers significant structural flexibility, thereby facilitating their destabilization. As shown in Figure 8d, this resembles common wool in daily life, which twists and bends into a disordered agglomeration due to the high aspect ratio and flexibility.^[^ ^45^, ^124^ ^]^ Destabilization results in a larger airflow section and a dominant drag force, with ULCNTs tending to wrap themselves into structurally more stable clusters of self‐similar characteristics.

In the growth environment of ULCNTs, this evolution rule of agglomerated structure will be induced by airflow disturbance and contain inherent physical mechanisms. Zhu et al.^[^ ^20^ ^]^ utilized magnets to manipulate substrate movement during CNT growth, altering speed and direction to cause micro airflow disturbances. This disruption results in local areas within the flow field deviating from stable laminar flow states toward unstable vortices. Figure 8e illustrates the self‐agglomeration structural evolution of CNTs during this process. It has been discovered that the curvature radius of fractal geometry in CNT tangles is entropy‐driven and determined monotonically by the relative tangential velocity difference in fluidic layers under the Kelvin‐Helmholtz instability.^[^ ^125^ ^]^ Under weak fluidic perturbation, an array of single‐mode point vortices aligned and remain stationary along the discontinuity surface,^[^ ^126^ ^]^ resulting in sinuous CNTs with neighboring peaks (*λ*) and horizontal displacement *ω*(*λ*) that increase with relative velocities between fluidic layers. As the relative velocities increase continuously, the mixing of each fluidic layer contributes to nonlinear paired vortices,^[^ ^127^ ^]^ resulting in a chaotic yet orderly patterned CNT which is affected by the bounded periodicity. It reflects the sinusoidal primary structure of a single CNT prior to entanglement formation. As the fluid disturbance persists, the interaction between primary structures induces entanglement on a larger scale, ultimately forming CNT tangles with hierarchical micro units. Based on experimental findings, all CNT structures exhibit limited horizontal displacement *δ*(*h*)_max_ that depends on the relative velocity Δ*v*, tangling length *L*, and gas velocity *U*, which can be described by δ(*h*)_max_≅0.2Δ*vL*/*U*. In addition, as the relative velocity increases in proportion to the Feigenbaum number,^[^ ^128^ ^]^ the *δ*(*h*)_max_ will be periodically multiplied, revealing the evolvement rule of the horizontal structure of CNT agglomerates.

Based on the interaction with the surrounding fluid environment, the self‐winding of ULCNTs caused by the gas velocity fluctuation reflects the alternating action of the drag force and the cohesive force of the gas flow, ultimately leading to the formation of entangled CNT structures. Figure 8f illustrates the spatial forces exerted on the self‐agglomeration structure of ULCNTs. For a floating tangled ULCNT, the gravity and thermal buoyancy reach an equilibrium along the vertical direction, while the evolution of the tangled structure is dominated by the velocity‐dependent drag force and size‐dependent viscosity force along the horizontal direction. With the increase of tangling length, the width *δ*(*h*)_max_ of the tangled structure increases linearly with gas velocity, so that the tangle will move forward under the interactions of drag force. When the viscosity force becomes large enough to impede its movement, part of the straight CNTs will be curved to increase the size of the tangle, then the drag force will increase with the airflow contact area so as to push the tangle forward. These fluidic behaviors dependent on the gas velocity and surface structure are quite similar to those grown CNTs in the vertical fluidized bed reactor. Therefore, we believe that the agglomeration of floating ULCNTs is an emerging dilute‐phase fluidization system, which belongs to generalized fluidization beyond the reactor form and direction of movement.

It should be noted that although we can put the agglomerated structures of ULCNTs into the Geldart phase diagram to explain their significance for the fluidization as Group‐C particles, we also believe that the traditional Geldart phase diagram is not enough to perfectly explain the horizontal agglomerated fluidization, which also requires more dimensional expansion of the Geldart phase diagram. Therefore, the horizontal agglomerated fluidization for ULCNTs can help endow classical fluidization with deep insights in contemporary times and indicate the necessity of developing new particle phase diagram theories with the rapid development of micro‐ and nano‐scale particles. Furthermore, based on the nano dilute‐phase fluidization science and technology, horizontal agglomerated fluidization is expected to push forward the mass production of those ULCNTs and related low‐dimensional nanomaterials in some high‐end applications such as CNT transistors and optoelectronics devices,^[^ ^124^, ^129^ ^]^ which will also contribute to better understanding the deep principles of nanomaterials self‐assembly in gas–solid catalytic reactions.

## 5 Fluidization Strategy of Composite Nanoparticles

### 5.1 Silicon‐Based Carbon Nanocomposites

**Figure 9:**
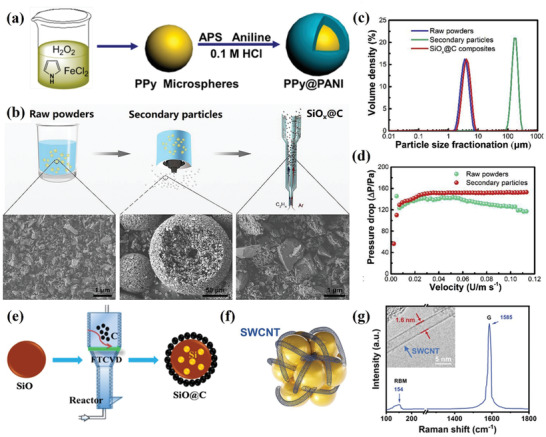
a) Schematic illustration of core‐shell composite particles prepared by solution polymerization. Reproduced with permission.^[^ ^130^ ^]^ Copyright 2015, The American Chemical Society. b) Schematic illustration of preparation to realize uniform coating of nano‐carbon on SiO_x_ nanoparticles. c) Particle size distribution curves of raw SiO_x_ powder, secondary particles, and SiO_x_@C composite. d) Fluidization curves of raw powder and secondary particles. b‐d) Reproduced with permission.^[^ ^141^ ^]^ Copyright 2019, Elsevier. e) Schematic of formation mechanism for carbon layer and Si crystal in the fluidized bed. Reproduced with permission.^[^ ^143^ ^]^ Copyright 2019, Elsevier. f) Schematic illustration of the contact mode between SiO_x_@C and SWCNTs. g) Raman spectrum revealed the perfect structure of SWCNTs. Inset, TEM image showing the diameter of an SWCNT. f,g) Reproduced with permission.^[^ ^144^ ^]^ Copyright 2023, Wiley‐VCH.

As an important form of realizing the transformation of nanoparticles into fluidizable forms, fluidization of nano agglomeration has achieved good results in the preparation of single component nano‐carbon materials such as various types of CNTs. The high efficiency of heat and mass transfer brought about by fluidization promotes stable chemical reactions, which has also attracted researchers' attention to the design of nanocomposite structures. In classic polymer particle design, nanomaterial composite structures are widely used for solution polymerization, which can form stable and uniform coating layers (**Figure** **9**a) and make good progress in organic–organic and organic–inorganic composite materials.^[^ ^130^, ^131^, ^132^ ^]^ Because in this design, composites can combine various functions of different materials and exhibit inherent properties that individual components do not possess. However, there has been relatively little progress in inorganic‐inorganic composite materials related to carbon, as carbon particles are usually difficult to dissolve in solution systems, limiting the efficient progress of the reaction. The gas–solid fluidization mechanism provides a clear strategy for regulating particle size and reaction uniformity, providing new design ideas for nano‐carbon composite particles. In early fluidization research, it was recognized that SiO_x_ is an excellent fluidization unit, and the development of nano agglomerate fluidization technology has also made its fluidization process stable and controllable. This also provides excellent carriers for supporting various nano‐carbon materials, especially graphene with single or multiple layers^[^ ^133^, ^134^ ^]^ and graphite nano‐carbon,^[^ ^135^, ^136^ ^]^ are often applied as shells for SiO_x_ cores to improve the electrical and thermal conductivities and protect the inner cores from unwanted chemical effects in the field of batteries. This kind of nanocomposite design can effectively reduce the side reactions between the electrode and electrolyte, and prevent cores from shape/morphology change, such as the huge volume expansion of the silicon anode in lithium‐ion battery (LIB) cycles.^[^ ^137^ ^]^

According to recent research, nano‐carbon materials can achieve self‐fluidization through agglomeration and can be combined with other difficult‐to‐fluidize materials as carriers or functional layers for the preparation and application of nanocomposites. For instance, nano‐silicon exhibits promising applications in batteries,^[^ ^137^ ^]^ but the minute diameter and extensive specific surface area make it prone to agglomerate during fluidization.^[^ ^31^ ^]^ Hence, achieving monodisperse particle preparation poses a challenge, and the performance may suffer seriously as a result of agglomeration. Composite with nano‐carbon materials as the coating layer provides a novel solution, resulting in particles with a widely adjustable diameter suitable for fluidization. This kind of nanocomposite also endows the nano‐silicon with excellent conductive properties, which helps to improve the stability and cycle life of the silicon negative electrode in the battery. However, carbon coating with excellent crystallinity and uniformity still poses two primary challenges. First, a perfect carbon shell can only be ensured by several strategies. Among these methods, CVD is widely utilized to obtain high‐crystallized carbon layers. Wu et al.^[^ ^138^ ^]^ effectively synthesized carbon‐coated anode materials for LIBs using the CVD method. They used Cu_3_Si as the core and catalyst, combined with dopamine self‐assembly to achieve particle agglomerate. Subsequently, ethylene was introduced to participate in the reaction, and a stable and highly crystalline carbon layer was formed. However, due to the reactor's non‐uniform heat and mass distribution, CVD as a fixed bed method often results in an uneven coating. Therefore, fluidized bed technology is a more suitable option. Compared to fixed bed reactors, fluidized bed reactors offer higher space velocities, resulting in more efficient gas‐solid contact and significantly improved mass and heat transfer efficiencies. They also prevent dead zone formation and ensure uniform carbon coating. However, achieving effective preparation while maintaining stable fluidization of bulk particles has long been a challenge that requires new optimization strategies.

### 5.2 Compound Expansion of Nano Agglomerations

As previously discussed, particle size is the primary determinant for material fluidization, and appropriate agglomeration size plays a crucial role in controlling fluidization state and reaction uniformity. A variety of nanocarbon composites can be obtained by fluidization strategies with different control methods. Coppey et al.^[^ ^139^ ^]^ investigated the fluidized bed chemical vapor deposition (FBCVD) method for silicon modification of MWCNTs. The uniform fluidization of CNT agglomerates enables silane to achieve surface deposition of silicon, resulting in a composite material suitable for electrochemical testing as LIB anodes. Shi et al.^[^ ^140^ ^]^ also utilized fluidization as a process strengthening method, introducing graphite particles to mitigate SiO particle binding, inhibit the growth of agglomerates, and enhance fluidization, ultimately achieving in situ growth of (SiO+G)/CNT composite materials. This strategy ensures the uniformity and stability of the CNT and graphite, and the 3D network structure effectively alleviates electrode expansion and improves the mechanical flexibility of the material. However, composite particles prepared by these strategies are typically doped mixtures that do not fully reflect fluidization advantages in the composite structure design. The requirements for cladding integrality and uniformity in nano carbon coating structure do not conflict with the fluidization mechanism. Agglomerate fluidization reactions involve material surface deposition and volume expansion processes that generate new surface structures, which can be viewed as a compound expansion process. The designed composite strategy not only facilitates the regulation of particle volume and coating thickness but also significantly enhances the performance of base materials.

The primary challenge in preparing nanocomposite structures lies in achieving stable fluidization of initial nanoparticles, which is also crucial for ensuring uniform carbon coating. For instance, commercial SiO_x_ particles are only ≈2 µm in size, making it difficult to attain a stable fluidized state necessary for successful carbon coating. Particle agglomeration design is a simple and efficient approach, and the key to this strategy is the agglomeration method based on hierarchical structure design, which is also defined as the agglomeration fluidized bed chemical vapor deposition method. The secondary particles formed through particle agglomeration offer a large specific surface area that facilitates effective gas‐solid reactions during fluidization. The external coating of each primary particle can be considered a compound expansion process, whereby the particle size satisfies the requirements for stable fluidization theoretically as the coating thickens. This expansion process is both flexible and controllable, representing a significant advancement in the preparation of inorganic‐inorganic composite particles through nano‐agglomerate fluidization. Xiao et al.^[^ ^141^ ^]^ utilized this approach for the mass production of SiO_x_@C spherical particles, which ensured effective heat and mass transfer within the particles by designing loose contact pomegranate‐like secondary particles. Figure 9b shows the process of uniform carbon coating on the surface of SiO_x_ nanoparticles. As the secondary particles are produced through spray granulation of a dextrin‐containing solution, the pyrolysis reaction of both dextrin and carbon sources occurs simultaneously. This results in SiO_x_@C returning to a singular particle state and evenly coating the carbon layer. Figure 9c illustrates the size distribution of particles during this process, while Figure 9d demonstrates the distinct fluidization behaviors of secondary particles. Compared with the raw powders, the secondary particles exhibit a significantly increased average size and a better fluidization characteristic. Their additional research^[^ ^142^ ^]^ revealed the gas‐solid phase regulation mechanism in the fluidization process, which guided the design of silicon oxide. The carbon layer deposition exhibited near‐layered growth and excellent electrochemical performance, achieving a 100‐kilogram‐scale pilot production. This large‐scale synthesis is attractive, and Xia et al.^[^ ^143^ ^]^ also synthesized high‐specific capacity SiO@C composite materials through fluidization thermal chemical vapor deposition, achieving kilogram‐scale preparation (Figure 9e).

In addition, agglomerate fluidization also provides various options for constructing different carbon coating structures, making it possible to improve the performance of composite materials further. Employing similar nano agglomeration compound expansion strategies, He et al.^[^ ^144^ ^]^ also achieved the integration of SWCNTs and SiO_x_ via fluidized beds (Figure 9f), constructing negative electrode materials with high initial Coulomb efficiency, stable cyclic, and high rate performances. As depicted in Figure 9g, SWCNT exhibits a perfect structure and can significantly improve the functionality of SiO_x_ particles. This represents a novel application of carbon nano agglomerate fluidization in material functional design. Furthermore, based on the principles governing agglomerate particulate fluidization, it is possible to investigate stable fluidization conditions for nanocomposite powders composed of other materials. Zhang et al.^[^ ^145^ ^]^ used FBCVD to prepare ultra‐coarse WC‐Co composite powders effectively and found that decreasing the deposition temperature can reduce the cohesion of deposited Co particles and effectively prevent defluidization. Increasing the particle size and gas velocity of WC enhances the collision force, which is conducive to fluidization. Overall, introducing the nano‐carbon composite strategy into the fluidization system not only enables the functionalization of single component particles but also facilitates the expansion of particles. This approach provides novel insights into physical fluidization behavior and chemical reaction behavior of functional particles, thereby stimulating further research in novel carbon nanoparticle agglomerate fluidization.

## 6 Summary and Outlook

**Figure 10:**
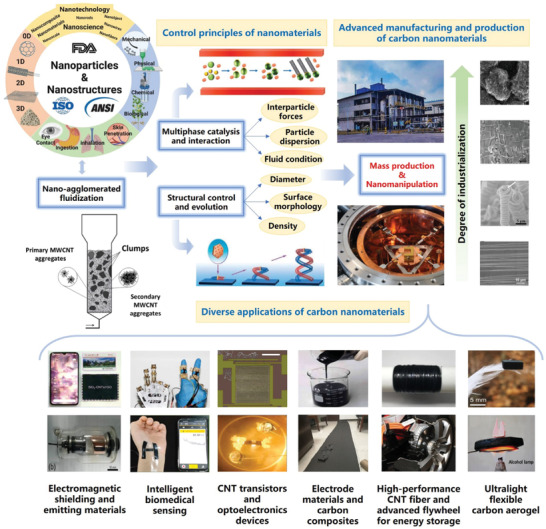
Control principles, advanced manufacturing, and applications of carbon nanomaterials and nano‐agglomerated fluidization emphasize the future development of nanoscience and emerging cross‐disciplinary fields. Reproduced with permission.^[^ ^32^, ^45^, ^97^, ^100^, ^101^, ^102^, ^109^, ^110^, ^111^, ^113^, ^114^, ^115^, ^116^, ^124^, ^129^, ^146^, ^147^, ^148^, ^149^ ^]^ Copyright 2022, 2008, 2009, 2021, 2010, 2012, 2023, 2018, 2022, 2015, 2021, 2022, 2017, 2016, 2008, 2022, 2021, 2023, 2017, Elsevier, Elsevier, Wiley‐VCH, Wiley‐VCH, Wiley‐VCH, The American Chemical Society, Springer, The American Chemical Society, Springer, Springer, Elsevier, The American Chemical Society, Optica Publishing Group, American Association for the Advancement of Science, The American Chemical Society, Multidisciplinary Digital Publishing Institute, Wiley‐VCH, The American Chemical Society, Springer.

Fluidization has long been a crucial research branch in particle reactions, playing an important role in chemical engineering, energy, and material engineering. Early research on fluidization was based on large‐sized particles, focusing on factors such as particle size, temperature, minimum fluidization velocity, and bed pressure drop. However, as the material size decreases to the nanoscale, the interaction between particles increases dramatically, placing them in Gelart's classification of difficult‐to‐fluidize particles. In order to facilitate the preparation of nanomaterials, agglomerate particulate fluidization has been developed and applied to various inorganic materials, particularly carbon nanomaterials, thereby enabling efficient heat and mass transfer processes. Nanomaterials represented by nano‐fibers, nano‐films, nano‐active materials, etc., will be promising for industrial production in the form of fluidization. Carbon nano agglomerations also deserve more extensive research because they represent the earlier success of nanomaterials to be massively produced, which will contribute to establishing industrial standards or quality systems for those nanomaterials with homogeneous physicochemical properties and stable nanoaggregate structures. **Figure** **10** summarizes the overall development logic from the nano‐agglomerated fluidization principle to mass production and nanomanipulation, and reflects the emerging cross‐disciplinary fields of carbon nanomaterials.

Agglomerate particulate fluidization is achieved by reasonably controlling the agglomerate structure of nanomaterials to transform them into fluidizable units rather than attempting to eliminate agglomeration phenomena. The construction of fluidizable units strongly relies on the surface structure of particles, intermolecular vdW force, and fluid velocity, which respectively represent the formation of internal cohesion and hierarchy of nano agglomerations, as well as the external interaction between nanoparticles and fluid. The synergistic effect of both factors facilitates the homogeneous and stable fluidization of nanomaterials within a fluidized bed, which holds significant implications for macroscopic synthesis. Given its potential application and the significant advantages of nano‐agglomerated fluidization, it is necessary to pay more attention and effort in the following areas:

1. Surface structure control promotes the formation of agglomerate fluidization. The rich surface morphology of nanomaterials implies various possibilities for control and modification. The loose secondary structure formed by the agglomeration of particles not only enables controllable fluidization but also facilitates efficient heat and mass transfer. This demonstrates excellent application value in preparing macroscopic CNTs and novel nanocomposites based on carbon nanomaterials. In addition, the multi‐stage structure design of CNTs can improve the specific surface area, strengthening the free‐molecular‐flow filtration of CNTs and reflecting the application potential of the air filtration field, which is of great significance to environmental science and human health.
2. Significant influence of fluid velocity on particle fluidization. The flow rate determines the interaction between particles and fluid, serving as an external regulatory mechanism. It not only affects pressure drop changes and chemical reactions in the entire system but also is a prerequisite for nano agglomerate formation. The strong cohesive force of nanomaterials often leads to the consolidation of the particle bed layer, and controlling the appropriate flow rate will break the bed layer and form fluidizable units. The selection and control of flow rate on fluidizable units is still worth further and extensive research.
3. An in‐depth understanding of the microscopic mechanism of nano‐agglomerated fluidization creates conditions for the development of new materials. At the nanoscale, 1D flexible materials represented by ULCNTs exhibit nano‐agglomeration behavior during dilute‐phase horizontal fluidization, further reflecting the hydromechanical interaction between gas flow and materials. In addition, the entanglement of an individual ULCNT transformed the hard‐to‐manipulate 1D material into an easily manipulated aggregate with a single chirality, which also provided new insights for the identification and separation of flexible low‐dimensional nanomaterials with high aspect ratios. Meanwhile, the structural design of nanocomposites further expands the research boundaries of nano‐agglomerated fluidization toward cutting‐edge advanced applications.
4. The nano‐agglomerated fluidization still keeps fresh and active in many emerging cross‐disciplinary fields. Only precise control over particle size and secondary structure will significantly avoid defluidization caused by out‐of‐limit agglomerations. Besides, it's important to pay more attention to developing novel fluidization technology and reactor equipment, so that those tough problems can be timely tackled in the material synthesis or assembly. For example, dilute‐phase nano‐fluidization is an innovative derivative that has now been widely used in floating catalyst CVD, typically exemplified by the preparation of carbon nanofibers, ULCNTs, etc. The dilute‐phase flow means domination of gas flow in the gas‐solid mixed system with less particle collision and lower probability of structural defects caused on the materials. In addition, concurrent‐up gas‐solid two‐phase flow is not the only reactor form for synthesizing high‐quality materials, radial reactors and downer reactors are also valuable platforms for the research of nano‐agglomerated fluidization in nanomaterials preparation.

Nano‐agglomerated fluidization is the scientific and technological basis of the mass production of materials. With the rapid development of new downstream application fields such as energy, electronics, and biomedical, nano‐agglomerated fluidization will motivate more fresh insights and vitality. It will establish a bridge linking basic science and cutting‐edge technology, which is expected to be fully developed in the mass production and emerging applications of nanocomposites, quantum materials, electronic materials, energy storage materials, metamaterials, etc.

## Conflict of Interest

The authors declare no conflict of interest.
